# American Medical Association

**Published:** 1848-06

**Authors:** 


### THE MEDICAL EXAMINER.

PHILADELPHIA, JUNE, 1848.

### AMERICAN MEDICAL ASSOCIATION.

The annual meeting of the American Medical Association, took place, according to adjournment, on Tuesday, the 2d ultimo, in the city of Baltimore, and consisted of about two hundred and fifty delegates, besides several surgeons of the army and navy, admitted by special resolution.

The Association met in the Universalist church, in Calvert street, at eleven o'clock, A. M., and was called to order by Dr. Chapman, the President elected last year.

The place provided by the committee of arrangements, (of which Dr. G. C. M. Roberts, of Baltimore, was chairman,) for the accommodation of the Association, was highly appropriate and convenient, and no effort was spared by the committee to facilitate the business of the meeting, and by the profession in the city to render the visit of their brethren from a distance interesting and agreeable :—the members may indeed be said to have been the guests of the city, so general were the invitations to visit the various interesting objects, procured for them by our kind brethren of the place, and so ample the hospitality exhibited in the entertainments given at their houses and at the several medical institutions.

Interesting discussions occurred on various matters pertaining to the business of the Association; and although often differing in opinion on the expediency of measures, it was abundantly manifest, that doctors, like all true gentlemen, could differ without disagreeing. Whatever other effects may result from the meeting, much kindly feeling was exhibited by the members one towards another; friendships were begotten, and prejudices in some instances removed, that for want of a better acquaintance and consequently truer appreciation of each other, might have endured through life.

After coming to order, and properly disposing of the minutes of the previous meeting, a committee consisting of one delegate from each State, was appointed by the chair, to nominate the several officers specified by the constitution, who reported the following, viz:

*President*—Dr. Alexander H. Stevens, of New York.

*Vice Presidents*—Dr. Samuel Jackson, of Philadelphia; Dr. John C. Warren, of Boston; Dr. Paul F. Eve, of Augusta, Georgia; Dr. William M. Awle, of Columbus, Ohio.

*Secretaries*—Dr. Alfred Stillé, of Philadelphia, and Dr. H. J. Bowditch, of Boston, Mass.

*Treasurer*—Dr. Isaac Hays, of Philadelphia.

From several of the standing committees *reports* were produced, which were read and referred to the Committee of Publication, viz: on Obstetrics, by Dr. Lindsly; on Surgery, by Drs. Norris and Parrish; on Medical Literature, by Dr. Holmes.

Dr. J. M. Smith, from the Committee on Practice of Medicine, having by an accident lost his report while coming to Baltimore, made a verbal statement of the topics it embraced. The report of this committee was likewise ordered to be presented to the Committee on Publication.

As these reports, with a full account of the proceedings of the Association, will shortly be published by the committee, we shall abstain from any remarks upon their character until we have them officially before us, when we shall transfer them to our pages, or, if too long, give abstracts of their contents. Some of them, it is likely, will afford occasion for remark, if not for dissent.

